# Splenic Infarct and Pulmonary Embolism as a Rare Manifestation of Cytomegalovirus Infection

**DOI:** 10.1155/2017/1850821

**Published:** 2017-10-11

**Authors:** Prashanth Rawla, Anantha R. Vellipuram, Sathyajit S. Bandaru, Jeffrey Pradeep Raj

**Affiliations:** ^1^Department of Internal Medicine, Monmouth Medical Center, Long Branch, NJ, USA; ^2^Texas Tech University Health Sciences Center, El Paso, TX, USA; ^3^Beth Israel Deaconess Medical Center, Harvard Medical School, Boston, MA, USA; ^4^Department of Pharmacology, St. John's Medical College, Bangalore, India

## Abstract

Cytomegalovirus (CMV) is a type of herpes infection that has a characteristic feature of maintaining lifelong latency within the host cell. CMV manifestations can cover a broad spectrum from fever to as severe as pancytopenia, hepatitis, retinitis, meningoencephalitis, Guillain-Barre syndrome, pneumonia, and thrombosis. Multiple case reports of thrombosis associated with CMV have been reported. Deep vein thrombosis or pulmonary embolism is more common in immunocompetent patients while splenic infarct is more common in immunocompromised patients. However, here we report a female patient on low-dose methotrexate for rheumatoid arthritis who presented with both pulmonary embolism and splenic infarct.

## 1. Introduction

Cytomegalovirus (CMV) is a type of herpes virus similar to herpes simplex, herpes zoster, or Epstein-Barr virus (EBV). It has a characteristic feature of maintaining lifelong latency within the host cell. Initial infection may just cause few symptoms, and some may shed the virus intermittently and asymptomatically, whereas, in immunocompromised individuals, reactivation may lead to a symptomatic disease. This resembles a mononucleosis-like syndrome with prolonged fever and hepatitis [1]. Thrombosis associated with acute CMV has many times been reported as various case reports ever since the 1980s [2]. However, here we report a rare case of thrombosis which involves both venous and arterial thrombosis of 2 different organ systems, namely, the lung as pulmonary embolism and the spleen as splenic infarct, respectively. To the best of our knowledge, using a PubMed search with MeSH terms (“cytomegalovirus” OR “CMV”) AND “thrombosis” did not reveal any case report that reported a patient who suffered from both splenic infarct and pulmonary embolism at the same time. Also, most cases of thrombosis are reported in patients in an immunocompromised state secondary to organ transplant or immune deficiency virus. We report a case of thrombosis in a rheumatoid arthritis patient on low-dose methotrexate.

## 2. Case Report

A 62-year-old female with a past medical history of rheumatoid arthritis for the last ten years on low-dose methotrexate (2.5 mg/week) was admitted with complaints of severe left upper quadrant abdominal pain. The intensity was rated as eight out of ten on a pain rating scale and was sharp and stabbing in nature. It was associated with nausea, but there was no vomiting. The patient also complained of diarrhea of 3–5 episodes per day over the last two months. The stool was watery, nonfoul smelling, large volume, not blood tinged, and devoid of mucus. The other concomitant medications included tablet folic acid 1 mg daily and tablet meloxicam 3.75 mg daily as needed for pain. She had no other comorbidities.

On clinical examination, there was no pallor, icterus, or lymphadenopathy. The temperature was 103°F with slight tachycardia. Her abdominal examination revealed moderate tenderness in the left upper quadrant with no guarding or rebound tenderness. Cardiac examination revealed no murmurs, and respiratory system examination was unremarkable.

At this juncture, a working diagnosis of acute abdomen was considered, and the investigations were directed to diagnose any common surgical causes. Her complete blood profile revealed a total white blood cell count of 6,200 cell/mm^3^ and a differential count of 4,340 neutrophils/mm^3^ (70%) and 930 lymphocytes/mm^3^ (15%). Platelets were 335,000/mm^3^, and the hemoglobin levels were 12.2 g/dl. Liver function tests revealed an ALT level of 73 UI/l, an AST level of 57 UI/l, an alkaline phosphatase level of 242 IU/l, and an LDH level of 298 UI/l. The C-reactive protein level was 49 mg/l.

**Figure 1:**
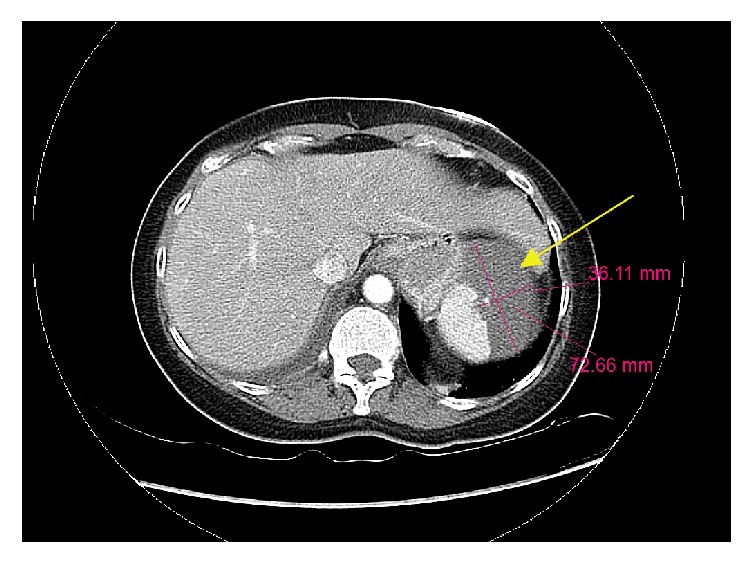
Computed Tomography (CT) of the abdomen done at admission with intravenous and oral contrast: the yellow arrow shows a high-density fluid filled large defect in the superior aspect of the spleen consistent with splenic infarct.

**Figure 2:**
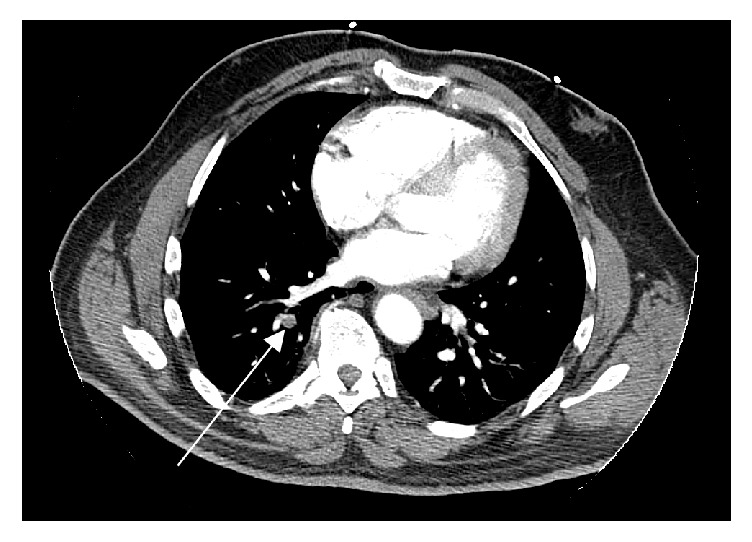
High-resolution CT scan of the thorax with intravenous contrast on day 2: the white arrow shows right lower lobe pulmonary embolism.

This was followed by a Computed Tomography (CT) of the abdomen done at admission, performed using intravenous and oral contrast. It showed a high-density fluid filled large defect in the superior aspect of the spleen consistent with splenic infarct (see Figure 1) and also a small pulmonary embolism in the right lower lobe. A high-resolution CT scan of the thorax with intravenous contrast confirmed the right lower lobe pulmonary embolism (see Figure 2). Lower extremity Doppler ultrasound showed no deep vein thrombosis. An echocardiography which was done revealed no valvular abnormalities.

The patient was started on anticoagulation therapy with rivaroxaban 15 mg per os (PO) twice daily. She was pan-cultured, and all the cultures including urine and blood were negative. Additional workup for chronic diarrhea including stool for* Clostridium difficile* and comprehensive stool cultures was all negative. Hematology-oncology was consulted for the hypercoagulable state. Protein C and S activity antithrombin 3, beta 2 glycoprotein and anti-cardiolipin antibody, and factor V Leiden mutation were all negative. Her liver function tests (LFTs) were abnormal. In light of elevated LFTs associated with fever and diarrhea, hepatitis virus, EBV, CMV, and Lyme titers were ordered. Hepatitis panel, EBV, and Lyme's test were negative. However, CMV immunoglobulin M (IgM) was 145 AU/ml (reference value: 0–29.9) and CMV immunoglobulin G (IgG) was 1.90 IU/L (reference value: 0.00–0.59). CMV quantitative PCR level was 43470 IU/ml (reference value: 200–2,000,000 IU/mL). CMV IgG avidity index was 0.46 (reference value ≥ 0.60). The patient was also tested for human immunodeficiency virus, and she was negative. She was started on valganciclovir with a loading dose of 900 mg twice daily for 14 days followed by a maintenance dose of 900 mg once daily for three months. Her diarrhea and abdominal pain started slowly getting better, and she had no more fevers. She was discharged on rivaroxaban for six months and valganciclovir. Repeat testing in 14 days for CMV IgM showed a level of 56.4 and CMV IgG level of 2.3. In 1 month, CMV IgM level was <30, CMV IgG was 5.01, and CMV quantitative PCR level was <200. Valganciclovir was discontinued after three months of treatment. At 3 months, CMV IgM level was <30, CMV IgG was 7.90, and CMV quantitative PCR level was <200. At 9 months, CMV IgM level was <30, CMV IgG level was >10, and CMV quantitative PCR level was <200.

## 3. Discussion

CMV manifestations can cover a broad spectrum from a little fever to as severe as pancytopenia, hepatitis, retinitis, meningoencephalitis, Guillain-Barre syndrome, pneumonia, and thrombosis [3]. CMV-associated thrombosis has been commonly reported in the literature and is independent of the other risk factors for thrombosis. In a retrospective study done by Atzmony et al. among 140 patients with acute CMV infection, the incidence of thrombosis was 6.4% (*n* = 9). Arterial thrombosis manifested as splenic infarcts (*n* = 4) and renal infarct (*n* = 1). Venous thrombosis presented as pulmonary embolism (*n* = 1), lower limb deep vein thrombosis (*n* = 1), upper limb deep vein thrombosis (*n* = 1), and jugular vein thrombosis (*n* = 1) [4]. However, a recent meta-analysis of 97 case reports of CMV infection associated thrombosis summarized that the majority (53.6%) of thrombosis cases occur as DVT/PE, which is followed by 25.8% in splanchnic veins and 12.4% as splenic infarcts. Also, DVT/PE is more common in immunocompetent patients while splenic infarct is more common in immunocompromised patients [5]. Other venous thromboses reported in the literature so far include internal jugular vein thrombosis, intracranial cerebral vein thrombosis, ovarian vein thrombosis, extrahepatic vein thrombosis, brachial vein thrombosis, and azygos vein thrombosis. Other arterial thromboses rarely include myocardial ischemia and digital ischemia [5]. Thus, it is interesting to note that our patient has suffered both PE and splenic infarct.

Multiple theories have been hypothesized on the role of CMV in causing thrombosis. The first is by the activation of factor X by enhancing platelets and leukocytes adhesion to infected endothelial cells. Another hypothesis is that CMV increases the circulatory levels of factor VIII. However, the most accepted theory is that CMV transiently induces the production of antiphospholipid antibodies (APLAs), which has been seen in several in vivo studies [6]. The pathophysiology of splenic infarcts included either CMV mononucleosis associated arterial insufficiency leading to rapidly enlarging spleen and resultant infarct or an arterial embolism [7].

The management of these patients included ruling out thrombosis in other sites. This is followed by initiation of antiviral agents like valganciclovir or ganciclovir and the use of an anticoagulation agent. So far, there has been no consensus on the choice of an anticoagulation agent and its duration, which is based mostly on the clinical decision of the treating physician [7, 8]. However, in our patient, anticoagulation therapy was initiated as the patient had venous thrombosis and we decided to continue the use of rivaroxaban at a dose of 15 mg PO twice daily for the initial 15 days followed by 20 mg PO once daily for six months.

## 4. Conclusion

Evaluation of CMV titers must be added to the diagnostic workup in the presence of a febrile splenic infarction or thrombosis especially when it is associated with a mononucleosis type of reaction. More research is warranted in this area before a routine diagnostic workup on thrombosis in cases of acute CMV could be advised. Thus, physicians should be more vigilant to look for complications of venous or arterial thrombosis in patients with acute CMV infection.
